# Massage Therapy for Fibromyalgia: A Systematic Review and Meta-Analysis of Randomized Controlled Trials

**DOI:** 10.1371/journal.pone.0089304

**Published:** 2014-02-20

**Authors:** Yan-hui Li, Feng-yun Wang, Chun-qing Feng, Xia-feng Yang, Yi-hua Sun

**Affiliations:** Department of Rehabilitation, Liaocheng People’s Hospital, Liaocheng, Shandong, China; University of Louisville, United States of America

## Abstract

**Background:**

Although some studies evaluated the effectiveness of massage therapy for fibromyalgia (FM), the role of massage therapy in the management of FM remained controversial.

**Objective:**

The purpose of this systematic review is to evaluate the evidence of massage therapy for patients with FM.

**Methods:**

Electronic databases (up to June 2013) were searched to identify relevant studies. The main outcome measures were pain, anxiety, depression, and sleep disturbance. Two reviewers independently abstracted data and appraised risk of bias. The risk of bias of eligible studies was assessed based on Cochrane tools. Standardised mean difference (SMD) and 95% confidence intervals (CI) were calculated by more conservative random-effects model. And heterogeneity was assessed based on the *I^2^* statistic.

**Results:**

Nine randomized controlled trials involving 404 patients met the inclusion criteria. The meta-analyses showed that massage therapy with duration ≥5 weeks significantly improved pain (SMD, 0.62; 95% CI 0.05 to 1.20; p = 0.03), anxiety (SMD, 0.44; 95% CI 0.09 to 0.78; p = 0.01), and depression (SMD, 0.49; 95% CI 0.15 to 0.84; p = 0.005) in patients with FM, but not on sleep disturbance (SMD, 0.19; 95% CI −0.38 to 0.75; p = 0.52).

**Conclusion:**

Massage therapy with duration ≥5 weeks had beneficial immediate effects on improving pain, anxiety, and depression in patients with FM. Massage therapy should be one of the viable complementary and alternative treatments for FM. However, given fewer eligible studies in subgroup meta-analyses and no evidence on follow-up effects, large-scale randomized controlled trials with long follow-up are warrant to confirm the current findings.

## Introduction

Fibromyalgia (FM) is a complex clinical syndrome characterized by chronic widespread pain and a cluster of other symptoms including anxiety, depression, sleep disturbance, stiffness, and a variety of somatic complaints [1]–[3]. Affecting 2% to 5% of the population, it has been identified as one of the most economically burdensome conditions [4]–[9]. Patients often seek symptomatic relief from multiple medical and complementary and alternative treatments [10]–[13]. And the research reported that around 90% of individuals with FM have used at least one form of complementary and alternative treatments to manage their symptoms [14], [15].

Massage therapy, as one of complementary and alternative treatments, has been widely used for FM. It may improve pain, anxiety, depression, and sleep disturbance by complex interplay of both physical and mental modes of action [16]. However, the conclusions of massage therapy for FM are inconsistent. Some reviews maintained that the effects of massage therapy for FM still kept inconclusive [17], [18] or negative [19]. The others suggested that the studies had provided beneficial evidence of use of massage therapy in treating FM [10], [20], [21], but most of them were only qualitative researches without a quantitative meta-analysis [10], [21]. In addition, traditional Chinese massage is one of the most ancient massage therapies, but most prior reviews did not include Chinese studies of massage therapy for FM due to language barrier or limited retrieving resources.

The objective of this systematic review and meta-analysis is to assess the evidence from recent English and Chinese randomized controlled trials (RCTs) on the effectiveness of massage therapy for FM, and evaluate whether massage therapy is a viable complementary and alternative treatment for FM.

## Methods

### 2.1 Search Strategy

The following electronic databases were searched from their inception to June 2013: PubMed, EMBASE, OVID-MEDLINE, SPRINGLINK, CNKI (China Knowledge Resource Integrated Database), Weipu Database for Chinese Technical Periodicals, and Wan Fang Data. The combination of the keywords included massage, manual therapy, Tuina, fibromyalgia, fibrositis, and myofascial pain. In order to include unpublished studies in our review, dissertations (ProQuest Dissertations and Theses A&I and Chinese Dissertation Full-text Database) and trial registration (Chinese Clinical Trial Registry) were also searched, and we also contacted experts in this field. Reference lists of retrieved articles were also screened to find any potentially relevant studies that had not been previously identified.

### 2.2 Study Selection

Only studies that met the following criteria were included: 1) RCTs of massage therapy for FM; 2) patients with FM were diagnosed according to the American College of Rheumatology criteria [3], and there were no limitations on the participant’s race, gender, or age; 3) massage therapy was viewed as an independent therapeutic intervention for FM, which did not combine with other manual therapies (chiropractic, mobilization, spinal manipulation, etc.); 4) any type of control interventions was eligible; 5) the main outcome measures were pain, anxiety, depression, and sleep disturbance, and no restrictions were set on the measurement tools used to assess these outcomes, because a large variety of outcome measures were employed in the studies; 6) the language was either English or Chinese.

### 2.3 Data Collection

Two reviewers (C Feng and X Yang) independently extracted data based on predefined criteria (Table 1). We contacted primary authors when relevant information was not reported. Differences were settled by discussion with reference to the original article. For crossover studies, we considered the risk for carryover effects to be prohibitive, so only the first phase of the study was selected in our review. We paid close attention to immediate (immediately after all treatments: up to one day) and follow-up effects of massage therapy for FM.

**Table 1:** Characteristics of randomized controlled trials of massage therapy for fibromyalgia.

First author,year, country	Sample size,Mean age (year)	Mean durationof fibromyalgia	Durationweeks	Follow-upweeks	Main outcomeassessments	Massage therapyintervention‡	Control group intervention
Sunshine	30	NR	5	–	Pain: VAS	Swedish massage	1)TENS
1996, US	50				Anxiety: STAI	(30 min/10 sessions)	2)Sham TENS
					Depression: CES-D		(30 min/10 sessions)
					Night difficult sleep		
Brattberg	48	NR	10	24	Pain: VAS	Connective tissue	No treatments or discussion
1999, Sweden	48				Anxiety: HAD	massage	
					Depression: HAD	(15 sessions)	
					Sleep: SDOS		
Alnigenis	37	8.6 years	24	–	Pain: VAS	Swedish massage	1)Standard care
2001, US	46				Anxiety: AIMS	(45 min/10 sessions)	2)Standard care and
					Depression: CES-D		telephone
Field	24	9.2 years	5	–	Pain: VAS	Swedish massage and	Progressive muscle
2002, US	51				Anxiety: STAI	Shiatsu	relaxation
					Depression: CES-D	(30 min/10 sessions)	(30 min/10 sessions)
					Sleep hours		
Lund	19	14 years	6	–	Pain: NHP	Massage therapy	Guided relaxation
2006, Sweden	51				Anxiety: CPRS-A	(30 min/12 sessions)	(30 min/12 sessions)
					Depression: CPRS-A		
Stiller	42	93% more than	1	–	Pain: VAS	Therapeutic touch	Placebo Mattress Pad
2006, US	51	2 years			Anxiety: STAI	(25 min/1 session)	(25 min/1 session)
Ekici	50	NR	3	–	Pain: VAS	Connective tissue	Lymph drainage therapy
2009, Turkey	38				Anxiety: FIQ	massage	(45 min/15 sessions)
					Depression: FIQ	(20 min/15 sessions)	
					Sleep: NHP		
Wang	90	12±4.4 months	3	–	Pain: VAS	Chinese traditional	1)Acupuncture
2010, China	40				Sleep: AIS	massage	(13 AP/20 sessions)
						(60 min/20 sessions)	2)Amitriptyline hydrochloride
							(30–50 mg/day)
Castro-Sánchez	64	NR	20	24	Pain: VAS	Massage myofascial	Disconnected Magnetotherapy
2011, Spain	48				Anxiety: STAI	therapy	(30 min/20 sessions)
					Depression: BDI	(90 min/20 sessions)	
					Sleep: PSQI		

NR = no reported; VAS = visual analog scale; STAI = state-trait anxiety inventory; CES-D = center for epidemiologic studies depression scale; TENS = transcut-aneous electrical nerve stimulation; HAD = hospital anxiety and depression scale; SDOS = sleep disturbance ordinal scale; AIMS = arthritis impact measurement scales; NHP = Nottingham health profile; CPRS-A = comprehensive psychopathological rating scale-affective; FIQ = fibrositis impact questionnaire ; AIS = Athens insomnia scale; AP = acupuncture points; BDI = beck depression inventory; PSQI = Pittsburgh quality of sleep index questionnaire.

‡ Massage therapy intervention/dose: number of massage time/number of sessions.

### 2.4 Quality Assessment

Two reviewers (C Feng and X Yang) independently assessed the risk of bias based on Cochrane tools in the following domains: random sequence generation, allocation concealment, blinding, incomplete outcome data, and selective outcome reporting [22]. We paid close attention to the blinding assessor in evaluating blinding methods because it is difficult to blind the therapists and patients for therapeutic methods in massage therapy studies. And the assessment of incomplete outcome data contained reporting dropout or withdrawal and intention-to-treat analysis. Every domain could be classified as “high” or “low” risk of bias. If the information reported in the paper was not sufficient, the domain was defined as “unclear”. There was no disagreement between the reviewers regarding quality assessments of eligible studies.

### 2.5 Data Analysis

The Cochrane Collaboration software (Review Manager Version 5.0) was employed in the meta-analyses. For continuous data, standardised mean difference (SMD) and 95% confidence intervals (CI) were calculated by more conservative random-effects model for the expected heterogeneity in the meta-analyses. Heterogeneity across studies was tested based on the *I^2^* statistic, a quantitative measure of inconsistency across studies, and studies with *I^2^*<40% was considered to have low heterogeneity, *I^2^* of 40% to 75% was considered moderate heterogeneity, and *I^2^*>75% was considered high heterogeneity. Detailed subgroup analyses were conducted based on different symptoms of FM and durations of massage therapy. Potential publication bias was assessed by visually inspecting of the Begg funnel plots.

## Results

### 3.1 Search Results

**Figure 1:**
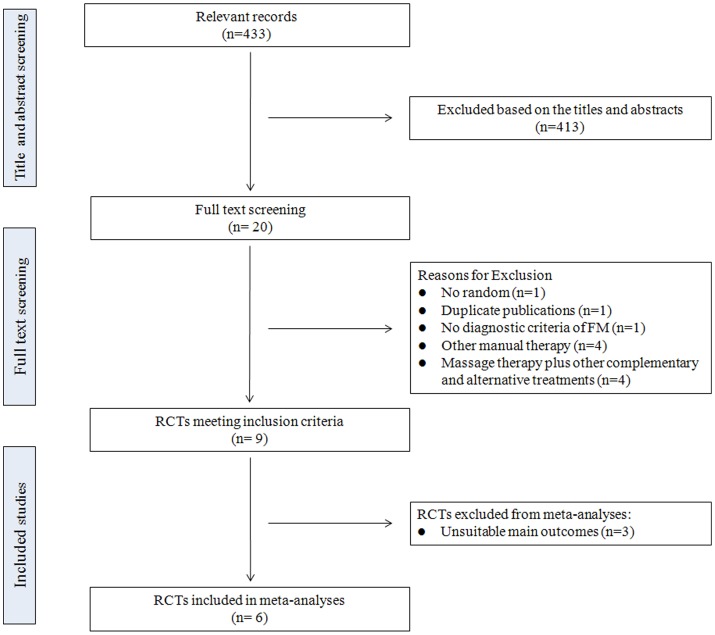
Flow chart for this meta-analysis. RCTs: randomized controlled trials; FM: fibromyalgia.

The initial search of the databases identified 433 relevant publications. After screening the titles and abstracts for inclusion criteria, 413 records were excluded based on various reasons including duplicate studies, reviews, commentaries, case report, not relevant to our analysis, and so on. In reviewing 20 full-texts, only 9 RCTs were considered for inclusion. 11 full-texts were excluded due to no random (n = 1), duplicate publications (n = 1), no diagnostic criteria of FM (n = 1), massage therapy plus other complementary and alternative treatments (n = 4), and other manual therapies (n = 4). In 9 eligible RCTs, 8 published in English [23]–[30] and 1 published in Chinese [31]. Six of them were included in meta-analyses [24], [26], [28]–[31]. The study selection process is depicted in Figure 1.

For Stiller’s study [28], the reviewers searched only the abstract, and got the dissertation by e-mail. And another study, the reviewers contacted the primary authors to get the mean and standard deviation (SD), because the author reported the results in figures [30].

### 3.2 Study Characteristics

The characteristics of 9 eligible RCTs are presented in Table 1. The studies were conducted in China, Spain, Sweden, Turkey, and US between 1996 and 2011. The sample size of eligible studies ranged from 19 to 90 (total 404, mean age of 47±4.87). The mean duration of FM ranged from 1 to 14 years. Massage therapy time lasted 25–90 min (total sessions ranged from 1 to 20). And the study duration ranged 1 to 24 weeks.

### 3.3 Methodological Quality

**Figure 2:**
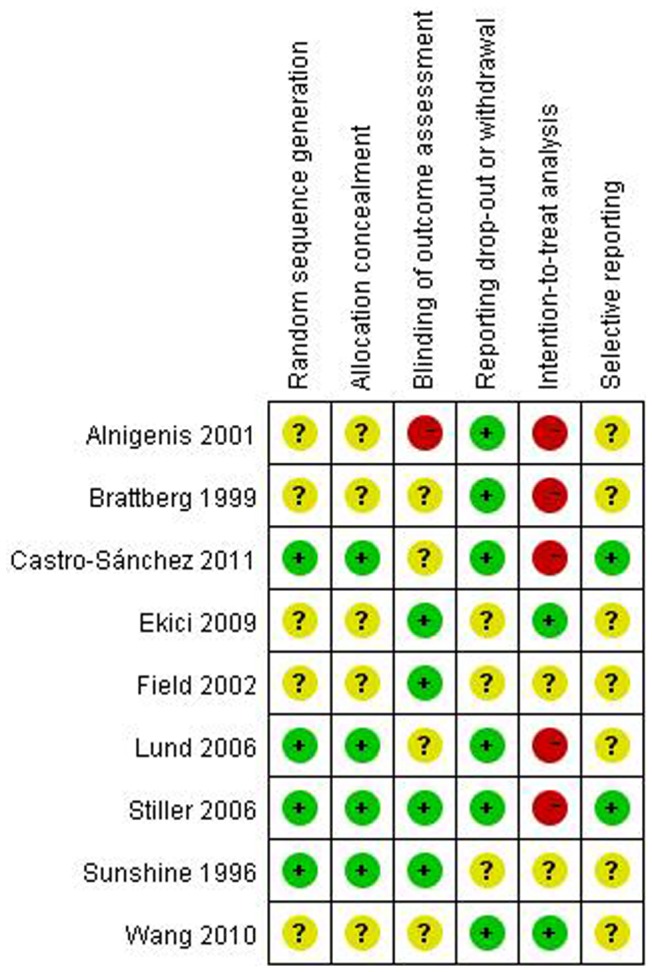
Risk of bias. Red (−): high risk of bias; Yellow (?): unknown risk of bias; Green (+): low risk of bias.

Risk of bias evaluation is reported in Figure 2. Four studies employed appropriate random sequence generation and allocation concealment [23], [27], [28], [30], others were unclear due to not state the detailed randomized and allocated method [24]–[26], [29], [31]. The authors reported that they employed blinded assessors in 4 RCTs [23], [26], [28], [29], while this parameter was unclear in four trials [24], [27], [30], [31], one failed to do so [25]. The risk of bias for reporting participant dropout or withdrawal was low risk in six RCTs [24], [25], [27], [28], [30], [31], others were unclear [23], [26], [29]. Five RCTs were high in the risk of bias for intention-to-treat analysis because the dropout data was cancelled. [24], [25], [27], [28], [30]. Two trials had a low risk of bias in the selective outcome reporting [28], [30], and others were unclear [23]–[27], [29], [31].

### 3.4 The Immediate Effects of Massage Therapy for FM

#### Pain

**Figure 3:**
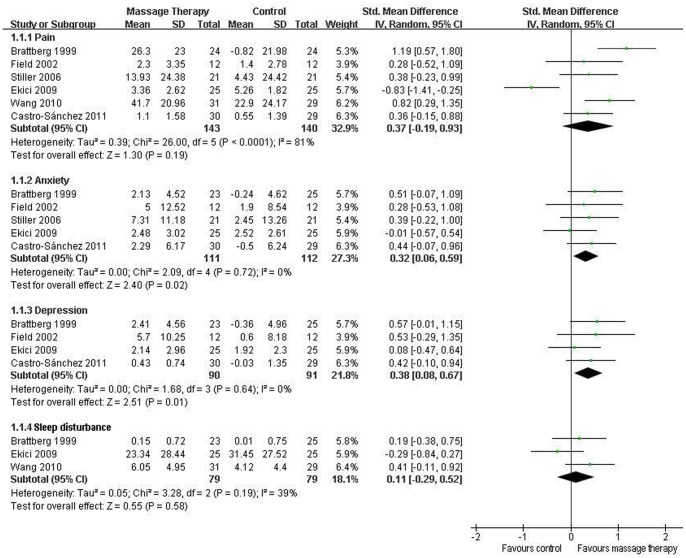
Forest plot showing the effect of massage therapy on pain, anxiety, depression, and sleep disturbance in patients of fibromyalgia.

**Figure 4:**
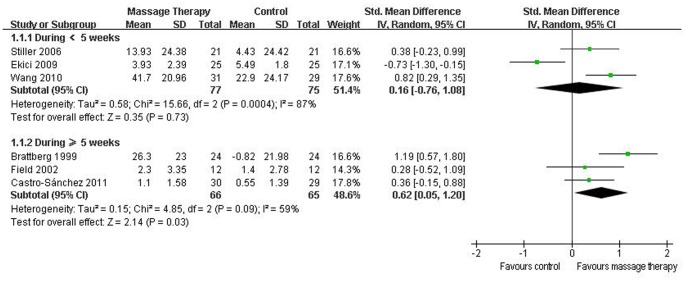
Forest plot of the subgroup analyses of massage therapy on pain in patients of fibromyalgia.

All nine RCTs reported pain. Six of them were included in the meta-analysis [24], [26], [28]–[31]. Although the aggregated results suggested that massage therapy was not associated with significantly reduced pain (SMD, 0.37; 95% CI −0.19 to 0.93; p = 0.19; Figure 3), massage therapy significantly reduced pain for duration ≥5 weeks (SMD, 0.62; 95% CI 0.05 to 1.20; p = 0.03; Figure 4) in subgroup analyses based on different durations.

#### Anxiety

**Figure 5:**
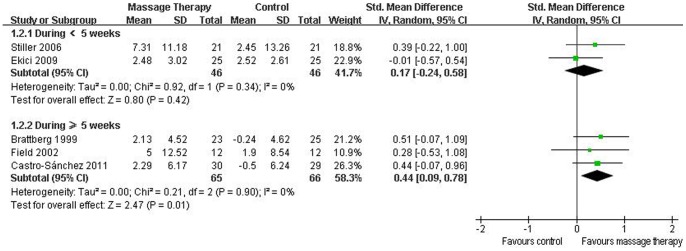
Forest plot of the subgroup analyses of massage therapy on anxiety in patients of fibromyalgia.

Eight RCTs evaluated the effectiveness of massage therapy on anxiety [23]–[30]. Five of them were included in the meta-analysis [24], [26], [28]–[30], which showed significant effects of massage therapy on anxiety (SMD, 0.32; 95% CI 0.06 to 0.59; p = 0.02; Figure 3). In the subgroup analyses, massage therapy did not improve anxiety for duration <5 weeks (SMD, 0.17; 95% CI −0.24 to 0.58; p = 0.42; Figure 5) [28], [29], but for duration ≥5 weeks, massage therapy significantly improved anxiety (SMD, 0.44; 95% CI 0.09 to 0.78; p = 0.01; Figure 5) [24], [26], [30].

#### Depression

**Figure 6:**
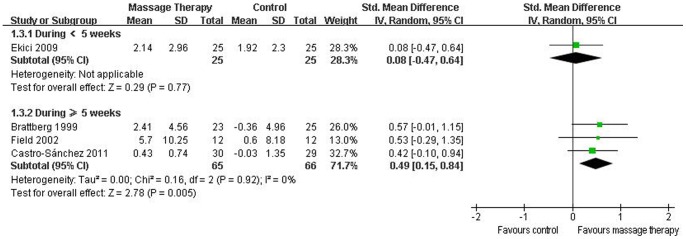
Forest plot of the subgroup analyses of massage therapy on depression in patients of fibromyalgia.

Seven RCTs assessed the effects of massage therapy on depression [23]–[27], [29], [30]. The results of the meta-analysis including 4 studies suggested that massage therapy significantly improved depression (SMD, 0.38; 95% CI 0.08 to 0.67; p = 0.01; Figure 3) [24], [26], [29], [30]. In the subgroup analyses, for duration <5 weeks, massage therapy did not reduce depression (SMD, 0.08; 95% CI −0.47 to 0.64; p = 0.77; Figure 6) [29], but for duration ≥5 weeks, massage therapy significantly improved depression (SMD, 0.49; 95% CI 0.15 to 0.84; p = 0.005; Figure 6) [24], [26], [30].

#### Sleep disturbance

**Figure 7:**
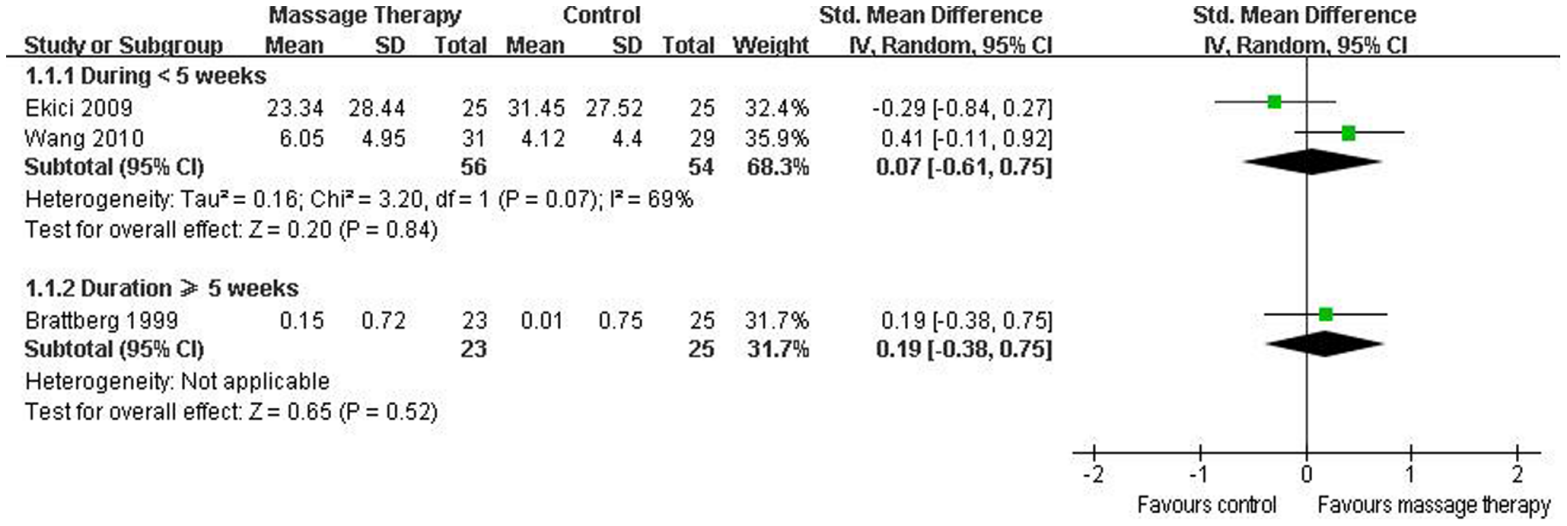
Forest plot of the subgroup analyses of massage therapy on sleep disturbance in patients of fibromyalgia.

Six RCTs reported the effectiveness of massage therapy on sleep disturbance [23], [24], [26], [29]–[31]. The aggregated results including three studies suggested that massage therapy did not significantly improve sleep disturbance (SMD, 0.11; 95% CI −0.29 to 0.52; p = 0.58; Figure 3) [24], [29], [31]. In the subgroup analyses, massage therapy did not significantly improve sleep disturbance for duration <5 weeks (SMD, 0.07; 95% CI −0.61 to 0.75; p = 0.84; Figure 7) [29], [31] or duration ≥5 weeks (SMD, 0.19; 95% CI −0.38 to 0.75; p = 0.52; Figure 7) [24].

#### Publication bias

**Figure 8:**
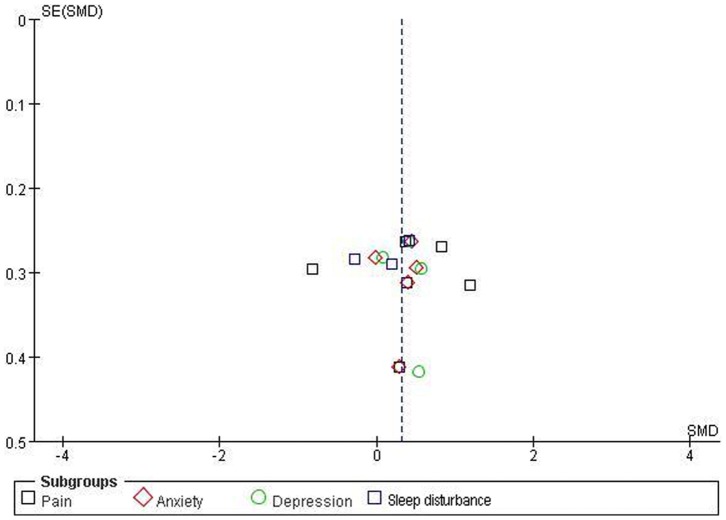
Funnel plot for pain, anxiety, depression, and sleep disturbance. Ernst E, Pittler MH (1997) Alternative therapy bias. Nature 385∶480.

The funnel plots for pain, anxiety, depression, and sleep disturbance were performed including 6 RCTs, 5 RCTs, 4 RCTs, and 3 RCTs, respectively (Figure 8), but it is difficult to interpret the result of publication bias due to such a small subset of studies.

### 3.5 RCTs Excluded in Meta-analyses

Three studies were excluded in meta-analyses due to incomplete data reporting. Sunshine et al reported that patients in massage therapy showed less pain, anxiety, depression, stiffness, and fewer nights of difficult sleeping [23]. The study conducted by Alnigenis et al did not show any significant benefit from massage therapy in alleviating pain, nor any effect on functional status and psychological distress in this small number of FM sufferers [25]. The study conducted by Lund found that pain and emotional reactions (including anxiety and depression) in patients with FM have decreased in all groups. But the comparison was not performed between the massage group and guided relaxation group on pain and emotional reactions [27].

### 3.6 The Follow-up Effects of Massage Therapy for FM

Only two RCTs assessed the follow-up effects of massage therapy for FM. At the end of 24 follow-up weeks, no statistically significant differences were observed for all the tested parameters compared with the baseline in the study conducted by Brattberg [24]. In Castro-Sánchez study [30], the experimental group showed significant improvements on pain, anxiety and sleep duration versus baseline after the 4 follow-up weeks. But at the end of 24 follow-up weeks, the experimental group only showed a significant improvement on sleep duration versus baseline.

### 3.7 Adverse Events

No adverse events were reported in all eligible RCTs of massage therapy for FM.

## Discussion

The purpose of our systematic review was to evaluate the evidence of massage therapy on improving symptoms in patients with FM. In immediate effects, our meta-analyses suggested that massage therapy with duration ≥5 weeks significantly improved pain, anxiety, and depression in patients with FM. There was no evidence that massage therapy showed superior effects on improving symptoms associated with sleep. In follow-up effects, there was not enough evidence of massage therapy for FM.

Our review was consistent with some prior reviews supporting use of massage therapy for FM. Kalichman conducted a qualitative review including 6 RCTs published from 1996 to 2009 and drew a general conclusion that there was modest support for use of massage therapy in treating FM [21]. The quantitative systematic review and meta-analysis performed by Kong et al. suggested that therapeutic massage may be helpful in the treatment of pain in patients with FM, but it was only a meeting abstract without the published full-text [20]. Comparing with these reviews, our systematic review updated the evidence of massage therapy for FM including more new RCTs published from 2006 to 2011 [28], [30]. And detailed subgroup meta-analyses were performed based on different symptoms of FM including pain, anxiety, depression, and sleep disturbance. In addition, we quantitatively assessed the effects of massage therapy with different durations for FM. Our results suggested that massage therapy with duration ≥5 weeks significantly improved pain, anxiety, and depression in patients with FM.

Our results differ from those of systematic reviews and guidelines [17], [18], [19]. Massage therapy was not recommended in the systematic review and guideline (6 RCTs from 1996 to 2009) performed in German [19]. And Terhorst and colleagues’ review (6 RCTs from 1996 to 2009) also suggested that massage therapy was not effective on pain relief in patients with FM [17]. One suspected reason for this difference was that more new RCTs of massage therapy for FM were included in our review [27], [28], [31]. And our review contained the study of Chinese traditional massage, as one of the most ancient massage therapies, for FM [31]. Furthermore, the meta-analysis included one study evaluated the effects of Tuina plus Yoga for FM in Terhorst’s review [17]. Another possible explanation was detailed subgroup meta-analyses were performed based on different durations of massage therapy in our systematic review. Although the overall aggregated result suggested that massage therapy was not associated with significantly reduced pain in our review, the subgroup meta-analysis supported that massage therapy with duration ≥5 weeks significantly improving pain in patients with FM. In addition, the mean change between final treatment and the baseline in outcomes was used to evaluate effect estimates of massage therapy versus controls. However, only outcomes at final treatment were employed in the meta-analyses by Germany systematic review [19]. More eligible RCTs and detailed subgroup meta-analyses strengthened our confidence in our review.

### 4.1 Possible Rationale of Massage Therapy for FM

If FM benefited from massage therapy, there might be a possible rationale derived form the complex interaction of both physical and psychological patterns. When massage therapy is delivered to soft and connective tissues, local biochemical changes would be stimulated. This helps to improve muscle flexibility, and modulate local blood and lymph circulation. As a result, local nociceptive and inflammatory mediators may be reabsorbed [32], [33]. Some studies found that massage therapy improved pain by modulating serotonin levels in patients with FM [34], [35]. The local effects may change neural activity at the spinal cord segmental level, which is responsible for both mood and pain perception [36]. Some studies maintained that massage therapy resulted in the reduction in the H-reflex [37]. A large reduction in the H-reflex would seem to be desirable, because spinal hyperexcitability is associated with a variety of chronic pain syndromes [38].

### 4.2 Study Limitations

There are several limitations in our review: 1) although we tried my best to make our search strategy to locate all relevant RCTs of massage therapy for FM, the degree of uncertainty may remain due to language barrier, limited retrieving resources, and publication bias [39,40]; 2) our review may also be affected by styles and dosing parameters of massage therapy such as styles (Swedish massage, Shiatsu, Chinese traditional massage, etc.), duration (time of each session), frequency (sessions of per week), and dosage (size of strength); 3) only RCTs were included and there were less eligible RCTs in some subgroups meta-analyses because of strict inclusion criteria in our review. Although it may influence the aggregated results, low inclusion criteria would result in more bias; 4) the results may be influenced by different outcome measures in eligible RCTs. So the reliable and valid outcome measures are essential to reduce bias, provide precise measures and perform valid data synthesis; 5) it is difficult to assess the duration of improvements on pain, anxiety, and depression in patients with FM because of fewer evidences in follow-up effects of massage therapy for FM. So long follow-up should be necessary in future RCTs of massage therapy for FM; 6) although the funnel plot for the outcomes were performed, it was difficult to interpret the results of publication bias due to a smaller subset of studies; 7) although no adverse events were associated with massage therapy, definite conclusions were not possible. It only can be assumed that massage therapy was a treatment option with low risk of injury.

## Conclusions

In conclusion, this systematic review found the positive evidence that massage therapy with duration ≥5 weeks had beneficial immediate effects on improving pain, anxiety, and depression in patients with FM. Massage therapy should be one of the viable complementary and alternative treatments for FM. However, given fewer eligible RCTs in subgroup meta-analyses and no evidence in follow-up effects, large-scale RCTs with long follow-up are warrant to confirm the current findings of massage therapy for FM.

## Supporting Information

Checklist S1 **PRISMA Checklist.**
(DOC)

## References

[1] Ge HY, Wang Y, Danneskiold-Samsoe B, Graven-Nielsen T, Arendt-Nielsen L (2010) The predetermined sites of examination for tender points in fibromyalgia syndrome arefrequently associated with myofascial trigger points. J Pain 11: 644–651. 19914876 10.1016/j.jpain.2009.10.006

[2] Hauser W, Eich W, Herrmann M, Nutzinger DO, Schiltenwolf M, et al (2009) The Fibromyalgia syndrome: classification, diagnosis, and treatment. Dtsch Arztebl Int 106: 383–391. 19623319 10.3238/arztebl.2009.0383 PMC2712241

[3] Wolfe F, Smythe HA, Yunus MB, Bennett RM, Bombardier C, et al (1990) The American College of Rheumatology 1990 criteria for the classification of fibromyalgia. report of the multicenter criteria committee. Arthritis Rheum 33: 160–172. 2306288 10.1002/art.1780330203

[4] Bannwarth B, Blotman F, Roué-Le Lay K, Carbère JP, André E, et al (2009) Fibromyalgia syndrome in the general population of France. A prevalence study. Joint Bone Spine 76: 184–187. 18819831 10.1016/j.jbspin.2008.06.002

[5] Branco JC, Bannwarth B, Failde I, Abello Carbonell J, Blotman F, et al (2009) Prevalence of fibromyalgia: a survey in five European countries. Semin Arthritis Rheum 39: 448–453. 19250656 10.1016/j.semarthrit.2008.12.003

[6] Bennett RM, Jones J, Turk DC, Russell IJ, Matallana L (2007) An internet survey of 2596 people with fibromyalgia. BMC Musculoskelet Disord 8: 27. 17349056 10.1186/1471-2474-8-27 PMC1829161

[7] Topbas M, Cakirbay H, Gulec H, Akgol E, Ak I, et al (2005) The prevalence of fibromyalgia in women aged 20–64 in Turkey. Scand J Rheumatol 34: 140–144. 16095011

[8] Thompson JM, Luedtke CA, Oh TH, Shah ND, Long KH, et al (2011) Direct medical costs in patients with fibromyalgia: cost of illness and impact of a brief multidisciplinary treatment program. Am J Phys Med Rehabil 90: 40–46. 20975520 10.1097/PHM.0b013e3181fc7ff3

[9] White LA, Birnbaum HG, Kaltenboeck A, Tang J, Mallett D, et al (2008) Employees with fibromyalgia: medical comorbidity, healthcare costs, and work loss. J Occup Environ Med 50: 13–24. 18188077 10.1097/JOM.0b013e31815cff4b

[10] Terry R, Perry R, Ernst E (2012) An overview of systematic reviews of complementary and alternative medicine for fibromyalgia. Clin Rheumatol 31: 55–66. 21614472 10.1007/s10067-011-1783-5

[11] Carville SF, Arendt-Nielsen S, Bliddal H, Blotman F, Branco JC, et al (2008) EULAR evidence-based recommendations for the management of fibromyalgia syndrome. Ann Rheum Dis 67: 536–541. 17644548 10.1136/ard.2007.071522

[12] Häuser W, Thieme K, Turk DC (2010) Guidelines on the management of fibromyalgia syndrome - a systematic review. Eur J Pain 14: 5–10. 19264521 10.1016/j.ejpain.2009.01.006

[13] Wang C, Schmid CH, Rones R, Kalish R, Yinh J, et al (2010) A randomized trial of Tai Chi for fibromyalgia. N Engl J Med 363: 743–754. 20818876 10.1056/NEJMoa0912611 PMC3023168

[14] Rossy LA, Buckelew SP, Dorr N, Hagglund KJ, Thayer JF, et al (1999) A meta-analysis of fibromyalgia treatment interventions. Ann Behav Med 21: 180–191. 10499139 10.1007/BF02908299

[15] Ernst E (2008) Complementary treatments in rheumatic diseases. Rheum Dis Clin North Am 34: 455–467. 18638686 10.1016/j.rdc.2008.03.007

[16] Imanura M, Furlan AD, Dryden T, Irvin EL (2012) Massage Therapy. In: Dagenais S, Haldeman S, editors. Evidence- based management of low back pain. Missouri: ELSEVIER. 216–28.

[17] Terhorst L, Schneider MJ, Kim KH, Goozdich LM, Stilley CS (2011) Complementary and alternative medicine in the treatment of pain in fibromyalgia: a systematic review of randomized controlled trials. J Manipulative Physiol Ther 34: 483–496. 21875523 10.1016/j.jmpt.2011.05.006

[18] Bronfort G, Haas M, Evans R, Leininger B, Triano J (2010) Effectiveness of manual therapies: the UK evidence report. Chiropr Osteopat 18: 3. 20184717 10.1186/1746-1340-18-3 PMC2841070

[19] Winkelmann A, Häuser W, Friedel E, Mooq-Eqan M, Seeqer D, et al (2012) Physiotherapy and physical therapies for fibromyalgia syndrome. Systematic review, meta-analysis and guideline. Schmerz 26: 276–286. 22760460 10.1007/s00482-012-1171-3

[20] Kong L, Bannuru R, Yuan W, Cheng Y, Fang M, et al.. (2011) Therapeutic massage on pain relief for fibromyalgia: a systematic review and meta-analysis. Arthritis Rheum 63(Suppl 10): 1917. [abstract].

[21] Kalichman L (2010) Massage therapy for fibromyalgia symptoms. Rheumatol Int 30: 1151–1157. 20306046 10.1007/s00296-010-1409-2

[22] Higgins JPT, Douglas GA (2008) Assessing risk of bias in included studies. In: Julian PTH, Green S, editors. Cochrane Handbook for Systematic Reviews of Interventions. Chichester, UK: Wiley-Blackwell. 187–241.

[23] Sunshine W, Field TM, Quintino O, Fierro K, Kuhn C, et al (1996) Fibromyalgia benefits from massage therapy and transcutaneous electrical stimulation. J Clin Rheumatol 2: 18–22. 19078022 10.1097/00124743-199602000-00005

[24] Brattberg G (1999) Connective tissue massage in the treatment of fibromyalgia. Eur J Pain 3: 235–245. 10700351 10.1053/eujp.1999.0123

[25] Alnigenis MN, Bradley JD, Wallick J, Emsley CL (2001) Massage therapy in the management of fibromyalgia: a pilot study. J Musculoskelet Pain 9: 55–67.

[26] Field T, Diego M, Cullen C, Hernandez-Reif M, Sunshine W, et al (2002) Fibromyalgia pain and substance P decrease and sleep improve after massage therapy. J Clin Rheumatol 8: 72–76. 17041326 10.1097/00124743-200204000-00002

[27] Lund I, Lundeberg T, Carleson J, Sönnerfors H, Uhrin B, et al (2006) Corticotropin releasing factor in urine-a possible biochemical marker of fibromyalgia. Responses to massage and guided relaxation. Neurosci Lett 403: 166–171. 16716515 10.1016/j.neulet.2006.04.038

[28] Stiller C (2006) The effect of therapeutic touch on fibromyalgia pain and anxiety. Case Western Reserve University (Health Sciences); Available: http://proquest.umi.com/pqdlink?did=1251875531& sid = 6&Fmt = 2&clientId = 17454&RQT = 309&Vname = PQD. Accessed 12 November 2009.

[29] Ekici G, Bakar Y, Akbayrak T, Yuksel I (2009) Comparison of manual lymph drainage therapy and connective tissue massage in women with fibromyalgia: a randomized controlled trial. J Manipulative Physiol Ther 32: 127–133. 19243724 10.1016/j.jmpt.2008.12.001

[30] Castro-Sánchez AM, Matarán-Peñarrocha GA, Granero-Molina J, Aguilera-Manrique G, Quesada-Rubio JM, et al.. (2011) Benefits of massage-myofascial release therapy on pain, anxiety, quality of sleep, depression, and quality of life in patients with fibromyalgia. Evid Based Complement Alternat Med. DOI: 10.1155/2011/561753. 10.1155/2011/561753 PMC3018656 21234327

[31] Wang J, Gao MX, Gao LQ, Tan ZD, Pan JY (2010) Clinical observation on 31 cases of fibromyalgia treated by traditional Chinese massage. Chin J Tradit Med Sci Technol 17: 72–73.

[32] da Silva GD, Lerenzi-Fiho G, Lage LV (2007) Effects of yoga and the addition of Tui Na in patients with fibromyalgia. J Altern Complement Med 13: 1107–1113. 18166122 10.1089/acm.2007.0615

[33] Goats GC (1994) Massage-the scientific basis of an ancient art: Part 2. Physiological and therapeutic effects. Br J Sports Med 28: 153–156. 8000810 10.1136/bjsm.28.3.153 PMC1332056

[34] Field TM, Sunshine W, Hernandez-Reif M, Quintino O, Schanberg S, et al (1997) Chronic fatigue syndrome: massage therapy effects on depression and somatic symptoms in chronic fatigue syndrome. J Chronic Fatigue Syndr 3: 43–51.

[35] Citak-Karakaya I, Akbayrak T, Demirtürk F, Ekici G, Bakar Y (2009) Short and long-term results of connective tissue manipulation and combined ultrasound therapy in patients with fibromyalgia. J Manipulative Physiol Ther 29: 524–528. 10.1016/j.jmpt.2006.06.019 16949941

[36] Sagar S, Dryden T, Wong RK (2007) Massage therapy for cancer patients: a reciprocal relationship between body and mind. Curr Oncol 14: 45–56. 17576465 10.3747/co.2007.105 PMC1891200

[37] Goldberg J, Sullivan SJ, Seaborne DE (1992) The effect of two intensities of massage on H-reflex amplitude. Phys Ther 72: 449–457. 1589464 10.1093/ptj/72.6.449

[38] Lidbeck J (2002) Central hyperexcitability in chronic musculoskeletal pain: a conceptual breakthrough with multiple clinical implications. Pain Res Manag 7: 81–92. 12185372 10.1155/2002/310974

[39] Egger M, Smith GD (1998) Bias in location and selection of studies. BMJ 316: 61–66. 9451274 10.1136/bmj.316.7124.61 PMC2665334

